# A qualitative study of perceived risk for HIV transmission among police officers in Dar es Salaam, Tanzania

**DOI:** 10.1186/1471-2458-13-785

**Published:** 2013-08-29

**Authors:** Edith AM Tarimo, Thecla W Kohi, Muhammad Bakari, Asli Kulane

**Affiliations:** 1 Department of Nursing Management, Muhimbili University of Health and Allied Sciences, Dar es Salaam, Tanzania; 2 Department of Internal Medicine, Muhimbili University of Health and Allied Sciences, Dar es Salaam, Tanzania; 3 Division of Global Health, Department of Public Health Sciences, Karolinska Institutet, Stockholm, Sweden

## Abstract

**Background:**

Understanding people’s views about HIV transmission by investigating a specific population may help to design effective HIV prevention strategies. In addition, knowing the inherent sexual practices of such a population, as well as the risky circumstances that may facilitate HIV transmission, is crucial for the said strategies to become effective. In this article, we report how police officers in Dar es Salaam, Tanzania, perceived the problem of HIV and AIDS in their local context, particularly in relation to unsafe sexual practices. The study was done with the view to recommending ways by which HIV transmission could be minimised within the police force.

**Methods:**

The study was conducted among members of the police force in Dar es Salaam, Tanzania. Eight focus group discussions (FGDs) were conducted, with a total of 66 participants who were mixed in terms of age, gender, and marital status. Some of these were caregivers to patients with AIDS. Data were analysed using the interpretive description approach.

**Results:**

The participants believed that both individual sexual behaviour and work-related circumstances were sources of HIV infection. They also admitted that they were being tempted to engage in risky sexual practices because of the institutional rules that prohibit officers from getting married during their training and for three years after. Nevertheless, as members of the Police Force, they stressed the fact that the risky sexual behaviour that exposes them to HIV is not limited to the force; it is rather a common problem that is faced by the general population. However, they complained, the nature of their job exposes them to road accident victims, subjecting them further to possible infection, especially when they have to handle these road accident casualties without proper protective gear.

**Conclusion:**

Individual sexual behaviour and job-related circumstances are worth investigating if proper advice is to be given to the police regarding HIV prevention strategies. In order to improve the lives of these police officers, there is a need to review the existing institutional rules and practices to accommodate individual sexual needs. In addition, improving their working environment may minimize the risk of HIV transmission from handling casualties in emergency situations.

## Background

Human Immunodeficiency Virus (HIV) and Acquired Immune Deficiency Syndrome (AIDS) remain a major public health concern that disproportionately affects sub-Saharan Africa (SSA) [1]. In 2009, the region had 12% of the global population, yet accounted for 69% of new HIV infections [1]. Behavioural interventions including promotion of comprehensive condom use have been underway to control HIV transmission, but for more than a decade the effectiveness of this intervention has been slowed down by insufficient behavioural change; unprotected sexual intercourse with multiple concurrent sexual partners remains the main problem [1]. A biomedical intervention, prevention of mother to child transmission, has been proven to decrease HIV transmission from mother to child, but inadequate access to antenatal and postnatal services remain a challenge [1]. Availability of a safe, affordable and accessible preventive HIV vaccine would be the best preventive measure. To date, a large number of phase I and II HIV vaccine trials have been conducted to assess the safety and immunogenicity of experimental vaccines, and a few phase III HIV vaccine trials have been conducted to assess the efficacy of the vaccine in preventing new HIV infections [2]. The only phase III trial that has shown modest efficacy is the RV144 trial conducted with thousands of Thai volunteers [3]. In view of the different circulating HIV-1 subtypes in different parts of the world, as well as the genetic differences of the populations, it will be necessary to conduct such trials in many different countries of the world.

Tanzania, with prevalent HIV-1 subtypes A, C, D [4] and an HIV prevalence of 5.1% in the age group of 15–49 years [5], is one of the countries in Africa conducting phase I and II HIV vaccine trials [2]. In 2007, Muhimbili University of Health and Allied Sciences (MUHAS) conducted a phase I/II HIV vaccine trial (HIVIS-03) with a candidate vaccine with matching circulating HIV-1 subtypes among volunteers from the police force in Dar es Salaam [6]. Members of the police force, like other uniformed services, are likely to engage in risky sexual practices [7]. An earlier study showed that police officers were engaging in multiple sexual relationships with low condom use [8]. Similarly, other studies from the general population in Tanzania have indicated that the main route of HIV transmission is unprotected heterosexual intercourse with multiple partners [9-12]. Since the conduct of a phase I/II HIV vaccine trial requires volunteers at low risk of getting HIV infection, we conducted an explorative study to assess what could hinder the police officers from enrolling in the HIVIS-03 trial. The results from the explorative study showed that potential volunteers were concerned about the effects of an experimental vaccine on their sexual practices, suggesting the possibility of the officers becoming less careful about their sexual behaviour during the experiment [13]. Data are presented here to illustrate how the police officers viewed the problem of HIV and AIDS, in their local context, particularly the problem of unsafe sexual practices as related to institutional practices.

## Methods

### Setting

The study was carried out in Dar es Salaam, Tanzania. Tanzania is situated in East Africa, immediately south of the equator, with an area of 947,300 square kilometres and a population of more than 48.2 million people, according to 2013 estimates. The Dar es Salaam region, located on the east coast of Tanzania, has an area of 1,393 square kilometres and an estimated population of 3.2 million people [14]. The region is divided into three municipalities: Temeke, Kinondoni and Ilala.

Thirty-two police stations serve these municipalities, which are both urban and peri-urban, and eight of these stations are represented in this study.

### Study population

This study was conducted among members of the police force in Dar es Salaam. It was a sub-study of the larger Sida/SAREC-funded Tanzania and Sweden (TANSWED) HIV Programme in Tanzania, which included studies of police officers such as HIV incidence, laboratory reference values, and related socio-behavioural issues. The programme facilitated the eventual conduct of a phase I/II HIV vaccine trial that enrolled 60 police officers [6]. Police officers were targeted for these studies because most of them have completed four years of secondary education, and they come from an established institution that facilitated access and follow-up of participating individuals. Taking into account the hierarchical nature of operations in the police force, one would have assumed some difficulty in ensuring confidentiality and volunteerism in HIV-related studies among the police officers. However, previous studies had shown that police officers can make independent informed decisions to take part in HIV-related studies that have support of higher police authorities [8,13,15].

### Study design and sampling

This was an explorative study conducted in a sub-group of police officers who had signed up for an HIV and AIDS education workshop [13]. Of the 408 police officers who had signed up for the workshop, 346 attended. The study participants were recruited from the workshop attendees a month after the workshop ended. These were recruited according to age, sex, rank, location of the police station, marital status, and whether the officer was taking care of a relative suffering from AIDS. The aim of recruiting persons who were caring for AIDS patients was to get first-hand information about the topic from people who had been directly affected. Therefore, the membership of focus groups had been predetermined; the 66 police officers were purposively chosen [16] to participate in the study (Table 1).

**Table 1:** Participants in the focus group discussion (FGDs)

**Group**	**Inclusion**	**Participants**
1	Young policewomen under or equal to 30 years old and unmarried (Young policewomen)	7
2	Older policewomen, above 30 years old, low rank; 3 of 4 were married (Older policewomen)	4
3	Low ranking policewomen, mixed age groups, 10 unmarried and 2 married (Low ranking policewomen)	12
4	Young policemen all under or equal to 30 years old and unmarried (Young policemen)	12
5	High ranking policemen, all above 30 years old and married	7
	(High ranking policemen)	
6	Low ranking policemen, all above 30 years old, half were married (Low ranking policemen)	6
7	Married and older policemen, mixed ranks	12
	(Married policemen)	
8	Police officers who have close contact with a relative with AIDS, 3 males and 3 females (Caregivers)	6
**Total**		**66**

NB: This table is accessible elsewhere [13].

### Data collection

Data were collected using focus group discussions (FGDs) [13]. The FGD guide was composed of general socio-demographic background information and posed two main research questions: (i) Can you tell me your views about the problem of HIV and AIDS in the police force? (ii) What are the cultural norms, views and opinions among police officers that could influence willingness to volunteer for an HIV vaccine trial? Results from the second question have been published elsewhere [13]; the present article focuses on the participants’ views on the problem of HIV and AIDS in the police force. Two field assistants, a doctor and a nurse from the police health unit, assisted in recruiting the participants. After the main researcher sorted out the names of the workshop attendees according to the inclusion criteria, the list was passed on to the field assistants. The field assistants in turn confirmed the availability of the potential participants, and the researchers elaborated on the study objectives.

All FGDs were conducted in private locations within the police stations that had been identified according to participants’ preferences. The number of participants varied between groups (Table 1). One of the main researchers moderated the discussions and took notes, assisted by a social scientist. Both manual and audio recording were done for backup. To complement participants’ responses, the note taker also documented non-verbal communication during discussion. The principles of confidentiality and privacy were adhered to and no individual names were mentioned on the audio tapes. Information collected was determined by the principle of ‘saturation’ [17]: enrolling new participants was stopped when the information started getting repetitive, and therefore enrolling additional participants seemed unhelpful in terms of getting new information. Duration of the discussion was between 25 and 71 minutes.

### Data analysis

The interpretive description (ID) approach was used, informed by principles of thematic content analysis [18] through an inductive design, to analyse the data. The audiotaped FGDs were transcribed verbatim and translated from Kiswahili to English. One of the main researchers, who is a fluent bilingual speaker of both Kiswahili and English, listened to the tapes and compared the Kiswahili version with the English version to ensure accuracy in transcription and translation. The information on the transcripts was inductively coded separately by two of the researchers and then discussed in order to increase the trustworthiness of the analysis. By using an inductive approach, the study sought to explore the data and identify emerging findings. Next, the researchers read and compared the codes and developed categories. In the process of developing categories, the main researcher interpreted the data and adjusted the categories slightly, staying close to the application potentials of ID [19]. The team discussed the preliminary findings with doctors, nurses, counsellors and the two field assistants. The use of two independent researchers in data analysis ensured reliability in interpretation while the discussion of preliminary findings with the research team brought additional insight into the interpretation.

### Ethical issues

Participants were recruited after approval had been granted by the Muhimbili University of Health and Allied Sciences (MUHAS) Institutional Review Board. The Police Authority also granted permission to conduct the study among its members. Before discussions started, informed consent was obtained from potential participants. The consent clearly described the principles of voluntary participation, anonymity, the right to withdraw without any consequences for the individual, and the need to audio record the discussions. Written consent, oral consent and review of the written consent with potential participants were completed to ensure that participants understood the implications of the consent and the meaning of voluntary participation.

## Results

### Participants

A total of 66 police officers participated in the study; of these, 35 were above 30 years old and the rest were between 19 and 30 years old. The mean age was 34 (SD = 10.2). Most participants were males (61%) and 56% were single (Table 1). The majority (85%) had had at least four years of secondary education.

### Theme and categories

During the analysis, one main theme about the views of HIV and AIDS among members of the police force emerged: ‘*Embracing risky sexual practices and unsafe work environment to rescue relationships’*. This theme, described in more detail below, refers to participants’ testimony about how they were engaging in risky sexual practices against institution rules to rescue sexual relationships. The theme also connotes participants’ obligation to save people’s lives in emergency situations without protective gear. The theme includes four categories as indicated in Table 2.

**Table 2:** Theme and categories

**Main theme**	**Embracing risky sexual practices and unsafe work environment to rescue relationships**
**Categories**	Engaging in risky sexual practices against institution rules
	Encountering risky sexual practices within and outside work place
	Detaching profession and ranks from sexual behaviour
	Compromising risk-taking practices to saving life

### Embracing risky sexual practices and unsafe work environment to rescue relationships

#### ***Risky sexual behaviour against institution rules***

During pre-service training and immediately after the training, participants became more aware of the fact that life is typically full of sexual desires, relationships, and risky sexual practices. They admitted that studying in the institutions for three to nine months had made it possible for men and women to interact more frequently. They believed that such interaction prompted establishment of sexual relationships between and among co-workers. Eventually, they said, this led to unprotected sexual intercourse causing sexually transmitted infections, including HIV. The participants who were caring for infected persons affirmed that education about HIV prevention is important to change trainees’ behaviour. They said that those in the institutions misbehave partly because of the institutional lifestyle. One participant explained:

“Truly, in the institutions, the behaviour [sexual practices] of rotating [multiple sexual relationships] among themselves [police officers] is very common. For example, a single male could be dealing with three women [sexual partners] at the same time…” (Participant 6, Group 8, Woman).

They added that many police officers who were HIV positive did not even know when they got the infection:

“The problem [HIV transmission] starts there [sexual practices]; who should bell the cat [who should warn another]? … You become infected even without knowing it…” (Participant 5, Group 8, Man).

They also admitted that many things were happening in the camps that could accelerate the spread of the disease:

“Wherever there is a gathering, these things [sexual practices] happen. We are living with different people in the camps, and temptations are many. Unprotected sexual intercourse contributes a lot in HIV transmission…” (Participant 2, Group 7, Man).

This typical sexual behaviour made it difficult for the police officers to adhere to institution rules that seemed to interfere with the officers’ sexual relationships and practices. Both men and women complained about the rule prohibiting them from getting married during pre-service training and within the three years that followed their training. Within these periods, the participants said that they established and broke off sexual relationships at will. Women in particular said that they were coerced to engage in multiple risky sexual practices because men did not want to use condoms, especially after an extended period of sexual relationship. Thus, women perceived themselves at higher risk of acquiring HIV infection due to such unprotected sexual intercourse. One woman said:

“You find a woman has stayed in a relationship for three years; I mean in a relationship with a man … the man may want to get married but the woman might tell him to wait for three years. As a result, the man ends the relationship and decides to look for another sexual partner. Three years of waiting is a long time but these are rules of the institution, and within this time, one may have many male sexual partners! Partners normally don’t bother to check each other’s HIV status, and some find it difficult to use a condom throughout their friendship…” (Participant 3, Group 1, Woman).

Another woman elaborated on the implication of the three-year rule:

“You may be in deep love with a man and agreed to wait for three years… Now, if you refuse to have sex [unprotected sexual intercourse] with him upon request, he goes out [engage in sexual relationship] with another woman. As a result, he may get infected with HIV, which he passes over to you” (Participant 5 Group 1, Woman).

Although some male officers were able to abstain from sexual intercourse during the pre-service training, they were not able to do the same after the training, during the in-service period. The newly employed officers, in particular, found the in-service rules difficult to observe. One participant clarified how the rule contributed to HIV transmission:

“This rule [in-service rule] requires young recruits to serve in the police force for at least three years before they get married… There are some people who are able to abstain [during pre-service], but they fail to do likewise when they start working. They engage in unprotected sexual intercourse. This [rule] contributes to HIV transmission…” (Participant 6, Group 4, Man).

Furthermore, the mandatory abstinence during pre-service training was reported to fuel risky sexual practices among newly employed police officers. One participant clarified on this:

“When they [newly employed police officers] report here [work station], they feel they have escaped from the depo [training institution] rules. … They start rushing here and there [having sexual intercourse with different women] because of the momentary freedom. By the time they make sense of their movements [risky sexual practices], they have already acquired the disease [HIV infection]” (Participant 1, Group 4, Man).

Other participants clarified the intention of the three-year service after the training, which they said is to enable newly employed officers to serve the police force for that period without interference of family responsibilities.

Also, sexual harassment and gender power imbalance appeared to fuel HIV transmission among newly employed women. Most women admitted that, after they had joined the service [after depo], they were approached by senior officers whose HIV status they did not know. They acknowledged the fact that young women were at high risk of contracting HIV because of having sexual relationships with senior officers. One of the participants gave this account:

“She [newly employed woman] might meet a senior officer who may approach her, and she might also give consent. This [relationship with senior officers] has made newly employed women avoid an HIV test because of the unsafe sexual practices in which they might have been engaged. You might find a person [woman] who has been on the job for only a month refusing to take the HIV test” (Participant 7, Group 1, Women).

Besides, female participants spoke of the complexity of dealing with men, especially on matters related to sex, when it comes to seniority and rank. They felt they would be risking their job if they refused sexual advances by senior officers. One of them elaborated on this problem:

“You may trick him [a senior officer], but when he finds out this was a trick, it creates bad feelings between the two of you. He will give you hard time, and also you may be fired from the job. Others just use their ranks and positions… and they [senior officers] normally become very persistent” (Participant 3, Group 1, Woman).

#### ***Encountering risky sexual behaviour within and outside the work place***

During the employment period, participants said that it was normal for male police officers to establish sexual relations with women police officers, including widows, who have been transferred in from other stations. However, being aware of the risk of HIV transmission, the men sometimes try to find out the HIV status of the widows. They added that it was important to find out the HIV status of the widows since such officers would be unsure of the cause of death of these husbands. One woman was not happy about women keeping as secret the cause of death of their husbands, especially if these men died of AIDS:

“My colleagues in Dar es Salaam [original workplace] might know that my husband died of AIDS. But when I am transferred to another workstation, nobody is going to know my status although it is written in the file [personal confidential file]. …” (Participant 1, Group 3, Women).

In addition, participants disclosed that when dealing with civilian cases, the male police officers sometimes solicit sexual favours or bribes from civilian women. Such officers might engage in unprotected sexual intercourse without being certain about the HIV status of the women. Participants clarified this situation thus:

“You find a civilian [woman] presenting a problem at the police station, and asking for assistance from a police officer [man]. The officer might get attracted to her and decide that he would not attend her for free [without negotiating for sexual favours]… the officer would normally propose to meet with the woman elsewhere [outside the workplace] after working hours” (Participant 1, Group 4, Man).

Another scenario suggested by participants was the fact that most of the police officers are involved in very tiring assignments, after which they need a bit of entertainment as a way to relax, often drinking alcohol in bars. They added that after drinking, the officers might be tempted to have unprotected sexual intercourse with barmaids without knowing their HIV status. They envisioned that in this way they might transmit the HIV infection to their spouses back home.

Another source of transmission of HIV that was pointed out by participants was working night shifts, where men and women work together in one shift. They said that although job rules do not allow sexual relations at the work place, this rule is sometimes ignored, especially when men and women officers are working in the same shift overnight and there is the possibility of engaging in unprotected sexual intercourse.

Further, participants blamed the system of relocation as one of the factors that contribute to HIV transmission among members of the police force, because members are forced to stay away from their families for a long time. One participant reported:

“As far as we are concerned [police officers] we are forced to work outside of our work stations for some time. For example, one may be deployed to a special operation [emergency duty outside one’s work station] for six months, away from one’s family for such a long time! In this kind of ‘separation’ chances of engaging in unsafe sex are high…” (Participant 4, Group 7, Man).

However, some older female participants were not convinced that transfers to different work stations or staying away from the families for a long time is a good enough reason to condone misbehaviour. They said that this kind of dishonesty is one of the main causes of HIV transmission within the community.

#### ***Detaching profession and ranks from risky sexual behaviour***

The kinds of behaviour the participants reported in this study are similar to risky sexual practices in the general population. In light of their experience, the participants noted that HIV infection is a disaster that has affected the whole society; and members of the police force are part of the society. They warned that it is not correct for civilians [non-police officers] to think that police officers are more vulnerable to the disease than anybody else in the society. They made this clear:

“You know, many people in the community do not know if a police officer behaves like them [civilians]… they think that police officers have supernatural powers; they are like angels; and that police officers may not fall sick, get tired or die… The police officers are like teachers, lecturers… same as doctors, nurses, security guards, housewives … Naturally, everybody has sexual desire. It is not correct to consider police officers as people who prefer ‘soft meat’ [sexual intercourse] compared to other people. Soft meat is a preference to everybody” (Participant 2, Group 5, Man).

Following the narrative above, all participants burst into laughter and started to talk to each other at random. By their non-verbal signals and body language, most of them seemed to agree to what the narrator had said.

One of the FGDs elaborated on the issue of sexual bribery, as exemplified in the following quote:

“Sexual bribery has affected the whole community. It is not uncommon to hear some female employee, e.g. a secretary complaining of something like: ‘My boss wants to have sex with me.’ … Truly, sexual bribery is rampant in the whole community, and not only among members of the police force” (Participant 9, Group 3, Woman).

One participant remarked that sexual bribery does not depend on rank. He said:

“It [sexual bribery] does not depend on rank or type of police officer. Any police officer, from lower to higher ranks; … all ranks are involved” (Participant 5, Group 4, Man).

Some participants believed that if police officers closely observed their duties and responsibilities they could prevent themselves from engaging in risky sexual practices, because their very tight schedules would keep them so busy that they wouldn’t have time to engage in risky sexual practices, as emphasised in the following quote:

“Here at the Field Force Unit (FFU), it is difficult to get involved in risky sexual practices because our activities force us rotate like robots [working continuously]. We are less affected, but in general, the problem [HIV transmission] exists” (Participant 1, Group 7, Man).

Others added some metaphorical expressions to describe the situation:

“For us in the force [police force], we have many activities; we have games, drill, etc. These kinds of open regular meetings reduce temptations [sexual temptations]. You may find someone joining the force [FFU] ‘sharp like a pencil’ [sexually active], but all over a sudden he changes into a ‘piece of chalk’ [less sexually active]” (Participant 2, Group 7, Man).

#### ***Compromising risk-taking practice to saving life***

In addition to engaging in risky sexual practices, members of the police force were also at risk of contracting HIV infection when attending to casualties. The police sometimes have to make difficult decisions whether to save the lives of such casualties without appropriate protective gear. Taking into account the expectations of the community, the officers said they have no choice except to do what is expected. This is evident from the narration below:

“We have been helping road accident victims and bullet-wounded casualties, with deep cuts… You may find this old man [pointing to a neighbour] boarding a police car to assist with the wounded, without gloves; not even simple plastic carrier bags that could be used instead of gloves. You find him touching the casualties unsure of their HIV status; and the community will obviously expect the police officers to remove the casualties or dead bodies from the accident site. This is the reality…” (Participant 3, Group 6, Man).

Although such cases were frequent, the police officers did not carry gloves with them, given the unpredictability of accidents. They said:

“Accidents occur every day and one does not expect a police officer to carry gloves all the time. You cannot predict accidents. One could only be told: ‘There has been a very bad accident; hurry up, go to the scene’… Without asking any questions, one sets out to try and save people’s lives, gloves or no gloves…” (Participant 8, Group 7, Man).

Participants pointed out that despite the fact that the police force has resources such as cars and doctors, the working environment did not ensure availability of these resources. They explained that in emergency situations it is difficult to manage the competing issues of fulfilling commands, availability and access to equipment, and the responsibility to protect the community in emergency situations:

“You are going around attending to your normal daily duties, and a car overturns in front of you. People are crying for help … you have to save people’s lives, by doing your best… After all, if you manage to save 20 lives and you die as a result, probably after coming into contact with contaminated blood, is your death justified or not?” (Participant 9, Group 7, Man).

Participants also pointed out that the police patrol car is normally equipped with all gear that might be required in an emergency. However, a police officer who is not on duty, probably on his/her way home, might find him/herself required to handle victims of an unexpected accident. In such a situation nobody expects this officer to have protective equipment. They are expected to prioritize saving people’s lives. One participant emphasised:

“Humanity in an accident scene overrides all protective procedure; somebody has been injured, and something has to be done” (Participant 8, Group 7, Man).

## Discussion

This study was undertaken among police officers to explore their views about the problem of HIV and AIDS in the police force. The researchers found that police officers are dissatisfied with the institutional rules that disallowed marriage and sexual relationships during pre-service training and during the first three years of service. Risky sexual practices among members of the police force during training may be a result of individual sexual desires and the institution lifestyle. One example of risky practices is the claim that senior officers approach young women for sex, which is contrary to the notion that senior officers are more responsible than those in junior positions, and would therefore not be expected to approach female officers for sexual favours. Mankayi [20] found out that female recruits who were studying at the same institution had intimate relationships with both undergraduate and postgraduate officers, implying that one’s sexual behaviour does not depend on seniority or administrative positions. Military authorities are against sexually oriented relationship among the different cadres [20] although the present study has shown that sexual relationships exist even during police training. This suggests that there has been laxity in enforcing the rule as practice has been shown to be inconsistent with the law.

The study has also shown that having extramarital sexual relationships among police officers is sometimes a result of spouses living away from each other; given their age, most of them are sexually active. They are therefore more likely to contract the disease than other age groups [21]. The risk of acquiring HIV is increased as a result of excessive consumption of alcohol among police officers. When people are intoxicated, they make poor judgement, including not using condoms during sexual encounters. One study has shown that among Nigerian naval personnel, respondents who had been posted abroad were significantly less likely to have used a condom, and more likely to have higher mean number of sexual partners [22]. Despite the women’s views that men should be faithful in their marriage while away, literature shows that there is a connection between job transfer, alcohol, and risky sexual practices that affects faithfulness towards spouses or other partners [22]. However, moving families from one station to another may not be practical because of unpredictable frequencies of relocations. In Nigeria, for example, 15% of police officers had been moved between five and ten times, and the majority of police officers experienced great difficulties in moving their families with them from one station to another; and those difficulties were associated with extramarital sexual relations and multiple sexual partners among the officers [23].

In addition, studies conducted among British military personnel in Belize and within the Canadian military have suggested that unsafe sexual behaviours should not be viewed as deviant or irrational. Instead, they should be seen as behaviours that are meaningful for the individuals concerned and that they conform to certain cultural ideals [24,25]. The unchanging sexual behaviour among the police officers in the present study may be exacerbated by deeply rooted traditional beliefs that encourage multiple sexual partners in contexts where the use of condoms is not favoured. Therefore, a specific intervention such as education could certainly be useful in changing people’s behaviour as suggested in a previous study among uniformed service personnel who thought of using condoms with casual partners only after an intervention [26].

The participants’ argument that the kind of sexual behaviour in question is shared by both police officers and the general public implies that risky sexual behaviour is a public health concern. It is therefore not surprising that a previous study among members of the police force in Dar es Salaam indicated that the HIV prevalence was comparable to that of the general population, and condom use was low [8], suggesting that both police officers and civilians to a great extent acquire HIV through the same risky behaviour. This notion is also supported by previous reports that have clearly shown that the primary mechanism for HIV transmission in Tanzania is unprotected heterosexual intercourse with multiple concurrent partners [9-12]. Similarly, the study among Nigerian naval personnel, mentioned earlier, shows that unprotected sexual contact with multiple partners was not only common among the naval personnel, but also among long distance drivers, traders, police officers, crew members and business men and women [22]. Likewise, in Nigeria and South Africa, police officers or soldiers had extramarital affairs with civilian women [20,23]. In the present study, the possibility of men engaging in sexual relationship with barmaids may be fuelled by the barmaids’ sexual behaviour. This assumption is supported by Yang [27], who found that women working in entertainment establishments engaged in risky sexual practices twice as frequently as those working in non-entertainment establishments.

The policies and programmes to address HIV/AIDS among peacekeepers and uniformed services, among others, underscore the following factors that may predispose members of uniformed services to contracting or transmitting HIV:

The members are predominantly young men and women who see themselves as invulnerable; duty schedules and periods of deployment result in separation from families; uniformed service personnel are often perceived by civilians as being privileged and in positions of power or authority; service men and women are more likely to have multiple partners and unprotected sex; condom use is incorrect or inconsistent; and service personnel tend to abuse alcohol and other substances [7].

On the contrary, the present study found that engaging in responsibilities and use of prevention methods may prevent participants from acquiring or transmitting HIV infection to others.

The police officers who participated in the FGDs believed that their risk of acquiring HIV was compounded by inadequate protection when handling casualties in emergency situations. Police officers’ exposure to such work-related risks is recognized among the factors which may predispose members of the uniformed services to contract or transmit HIV [28]. Thus, improvement of the working environment for police officers is required to minimize work-related risks of HIV transmission.

### Limitations

The study was conducted with a small sample, so generalization of the findings would be limited to the studied population. Interpretation of the ways of speaking among the study participants demanded attention of a native speaker. In the present study, participants often seemed to be speaking in riddles and metaphors, giving examples like ‘sharp pencil and chalk’ that required a native speaker’s interpretation. The first author is a native speaker, born and living in Tanzania, and so was able to deal with the language elasticity, in African contexts, as referred to by Airhihenbuwa [28].

## Conclusion

Given the findings from this study, there are a number of recommendations, which need to be taken into account for the successful prevention of HIV transmission among members of the police force. Risky sexual behaviour may be dealt with at the individual level through education and consistent counselling. Additionally, implementation of strategies such as reviewing institution rules to accommodate individual sexual needs may minimize risks of acquiring HIV infection. The availability of and access to protective equipment during emergencies may minimize the risk of acquiring HIV infection from road accident or crime-related casualties.

## Competing interests

The authors declare that they have no competing interests.

## Authors' contributions

EAMT conceived the study, coordinated data collection, carried out analysis and drafted the manuscript. AK participated in the study design, data analysis and reviewed the first draft of the manuscript. TWK and MB were involved in study design and critically reviewed the final draft of the manuscript. All authors read and approved the final manuscript.

## Pre-publication history

The pre-publication history for this paper can be accessed here:

http://www.biomedcentral.com/1471-2458/13/785/prepub
